# Post-Operative Pain After Endodontic Instrumentation, Irrigation and Obturation: An Umbrella Review of Systematic Reviews Published from 2016 to 2025

**DOI:** 10.3390/jcm15124775

**Published:** 2026-06-19

**Authors:** Fausto Zamparini, Andrea Spinelli, Gioia Quadrini, Maria Giovanna Gandolfi, Carlo Prati

**Affiliations:** 1 Endodontic Clinical Section, Department of Biomedical and Neuromotor Sciences (DIBINEM), Dental School, University of Bologna, 40125 Bologna, Italy; fausto.zamparini2@unibo.it (F.Z.); gioia.quadrini@studio.unibo.it (G.Q.); carlo.prati@unibo.it (C.P.); 2 Laboratory of Green Biomaterials and Oral Pathology, Department of Biomedical and Neuromotor Sciences (DIBINEM), Dental School, University of Bologna, 40125 Bologna, Italy; mgiovanna.gandolfi@unibo.it

**Keywords:** postoperative pain, endodontics, root canal therapy, umbrella review, meta-analysis

## Abstract

**Background:** The objective was to synthesize and critically appraise systematic reviews with meta-analysis evaluating the association between irrigation, instrumentation, and obturation procedures and post-operative endodontic pain. **Methods:** An umbrella review was conducted following PRISMA guidelines. Electronic searches identified systematic reviews published between 2016 and 2025. Eligible studies are systematic reviews that include meta-analyses, published in English and correlating the presence of post-operative pain in 3 different critical stages of root canal treatments, namely irrigation, instrumentation and obturation. Methodological quality was assessed using the AMSTAR 2 tool. Outcomes included pain prevalence and intensity at different time points. **Results:** Out of 368 records, 25 systematic reviews with meta-analysis met the inclusion criteria: 9 on irrigation, 8 on instrumentation, and 8 on obturation. NaOCl concentrations, irrigant activation, and intracanal cryotherapy were repeatedly reported as being associated with reduced short-term post-operative pain. For instrumentation, most reviews reported lower pain with rotary systems, but two studies found no difference or favored reciprocating kinematics. Apical patency did not appear to increase pain and foraminal enlargement may increase early pain. No clinically consistent differences were observed between bioceramic/calcium silicate-based and resin-based sealers, although calcium silicate sealers seem to support periapical healing. However, the certainty of these findings was limited by heterogeneity, methodological weaknesses, and overlap among primary studies. Methodological limitations were identified across reviews, mainly related to no protocol registration (n = 4), incomplete reporting of excluded studies with justification (n = 11), limited assessment of publication bias, and poor reporting of funding sources for primary studies. **Conclusions:** Based on current evidence, irrigation, instrumentation, and obturation procedures may influence short-term post-operative pain. However, these findings remain tentative because of heterogeneity, methodological weaknesses, variable review quality, and overlap among primary studies. Further high-quality reviews and clinical trials are needed.

## 1. Introduction

Post-operative pain (POP) is a common and clinically relevant outcome following endodontic treatment, with reported prevalence ranging from mild discomfort to severe pain requiring analgesic intervention [1,2,3]. POP after endodontic treatment is multifactorial and may be influenced by several patient-related, anatomical and biological variables, including preoperative pain status, pulpal and periapical diagnosis, systemic health, immune response, psychological factors, medication intake and occlusal status [2].

However, POP related to root canal treatment may also depend on treatment-related events, such as apical extrusion of the endodontic infected smear layer, irrigant solutions, obturation material, and instrumentation beyond the apical limit [1,2,3].

Despite significant advancements in instruments, materials, and clinical protocols, a considerable proportion of patients still experience POP after root canal therapy [1]. This symptom may negatively affect patient satisfaction and perceived quality of care, and quality of life overall, emphasizing the clinical relevance of effective strategies for pain prevention and management [4].

Several clinical variables have been investigated for their potential role in modulating POP. These include the type and concentration of irrigants, irrigation delivery systems, instrumentation techniques, apical preparation size, obturation materials, and use of intracanal medicaments or adjunctive strategies [3,5,6].

Over the past 10 years, endodontics has experienced significant technological and procedural advancements, including the introduction of bioceramic materials [7,8,9], reciprocating instrumentation systems [10,11,12,13,14], novel irrigation strategies and activation techniques [15,16]. These innovations have substantially modified clinical protocols and may influence periapical tissue response and the incidence of post-operative pain [17,18,19]. A high number of randomized clinical trials and systematic reviews have explored whether modifications of these parameters can reduce the incidence and severity of post-operative pain [1,2,3]. The available evidence remains, unfortunately, fragmented.

Systematic reviews and meta-analyses often report inconsistent findings, partly due to differences in study design, pain assessment methods, follow-up durations, and methodological quality [1,2]. This substantial heterogeneity among reviews limits the ability to draw definitive clinical recommendations.

Umbrella reviews may offer a comprehensive framework for evidence synthesis by integrating and critically appraising findings from multiple systematic review and meta-analysis. In endodontics, only few recent umbrella reviews have been published focusing on the growing use and documentation of new hydraulic sealers and their relation with POP [20]. Critical gaps emerge when we consider the instrumentation and irrigation protocols related to post-operative pain, as these phases are highly related to POP and other frequent complications (i.e., flare-up).

Therefore, the aim of this umbrella review was to synthesize evidence from systematic reviews and meta-analyses published over the past 10 years regarding post-operative pain associated with three procedural phases of endodontic treatment: irrigation protocols, instrumentation techniques, and obturation strategies.

## 2. Materials and Methods

### 2.1. Study Design, Protocol Registration and Reporting Guidelines

This study was conducted as an umbrella review of systematic reviews and meta-analyses evaluating post-operative pain following endodontic treatment. The analysis was made in accordance with the Preferred Reporting Items for Systematic Reviews and Meta-Analyses (PRISMA) guidelines [21] and following the recommendations for umbrella reviews proposed by Choi et al. 2023 [22]. The study protocol was uploaded and registered in PROSPERO (Protocol CRD420261331928 on 6 March 2026).

### 2.2. Research Question

The objectives of the literature review were summarized in the following research question, formulated according to the Population–Intervention–Outcomes–Study design (PIOS):

“What is the available evidence, derived from systematic reviews and meta-analyses, regarding the prevalence, intensity, and factors associated with post-operative pain in patients undergoing non-surgical endodontic treatment?”

Population (P): Patients undergoing non-surgical endodontic treatment;
Intervention (I): Endodontic treatment, techniques, or clinical strategies aimed at preventing or reducing post-operative pain (instrumentation, irrigation, obturation);
Outcome (O): Intensity and duration of post-operative pain;
Study (S): Systematic reviews and meta-analysis.

### 2.3. Eligibility Criteria and Information Source

In this umbrella review, we decided a priori to include only systematic reviews with meta-analysis to focus on quantitatively synthesized evidence and improve comparability across procedural domains.

Reviews were eligible on the basis of the following criteria:

- Systematic reviews of in vivo clinical studies on patients undergoing non-surgical endodontic treatment (timeframe 2016–2025).
- Published in English.
- With a dental/endodontic focus, identified through database-specific dentistry categories where available and through topic-based eligibility screening.
- Investigating the relationship between the presence of post-operative pain and the main steps of root canal treatment (irrigation solution, instrumentation, and obturation) in permanent teeth affected by various pathological conditions.

Reviews that did not meet the inclusion criteria were excluded, such as:

- Narrative reviews;
- Scoping reviews;
- In vitro studies;
- Systematic reviews on primary dentition;
- Systematic reviews concerning only pulp capping treatments;
- Systematic reviews lacking a comparative group.

### 2.4. Search Strategy

An electronic search was performed in PubMed/MEDLINE, Scopus, and Web of Science to identify systematic reviews and meta-analyses.

The strategy was structured using predefined conceptual blocks combined with the Boolean operator AND. Within each block, synonyms and related terms were combined using OR. The main conceptual blocks included: pain-related terms, endodontic treatment terms, procedure-related terms, and study-design terms related to systematic reviews and meta-analyses.

Our search strategy included both controlled vocabulary terms and free-text terms. MeSH terms included, among others, “Pain,” “Pain, Postoperative,” “Endodontics,” “Root Canal Therapy,” “Dental Pulp Diseases,” “Periapical Diseases,” “Root Canal Irrigants,” “Sodium Hypochlorite,” “Chlorhexidine,” “Root Canal Filling Materials,” and “Dental Instruments.” Free-text terms were expanded to include broader pain-related synonyms, such as “postoperative pain,” “post-operative pain,” “postendodontic pain,” “post-endodontic pain,” “post-obturation pain,” “interappointment pain,” “flare-up,” “analgesic consumption,” and “symptomatic apical periodontitis.” Procedure-related terms included irrigation, irrigant activation, sodium hypochlorite, chlorhexidine, cryotherapy, instrumentation, rotary and reciprocating systems, apical patency, obturation, sealers, bioceramic materials, calcium silicate-based sealers, epoxy resin-based sealers, gutta-percha, and intracanal medicaments.

For Scopus and Web of Science, the search strategy was adapted using database-specific field tags and equivalent free-text terms. Searches were restricted to publications from 2016 to 2025 and to articles published in English. Where available, database filters and subsequent topic-based eligibility screening were used to retain records with a dental/endodontic focus (Table S1).

An additional manual search was performed in relevant endodontic and dental journals, including the *International Endodontic Journal*, *European Endodontic Journal*, *Journal of Endodontics*, *Clinical Oral Investigations*, and *Australian Endodontic Journal* to identify any additional eligible systematic reviews and meta-analyses not retrieved through the electronic database search.

### 2.5. Study Selection

Two reviewers independently screened titles and abstracts for eligibility. Full texts of potentially relevant articles were retrieved and assessed independently. Disagreements were resolved through discussion or consultation with a third reviewer.

At first, duplicate records were removed, and titles and abstracts were screened. In the second stage, full-text assessment of potentially eligible articles was performed.

### 2.6. Data Extraction and Collection

Data was extracted by 2 independent reviewers using a standardized form. The form was discussed among the reviewers and with one expert operator included as the Author. Before data extraction, the reviewers performed a pilot calibration test. The search outputs and the data extraction were tested using potentially eligible reviews. The reviewers, expert endodontic operators, independently completed the extraction form, compared the extracted data, and refined the form where needed to ensure consistent interpretation of the extracted variables.

The following information was collected: the first author’s name, year of publication, pain-related factor, sample size/unit reported (number of patients or treated teeth), indication for endodontic treatment, publication period of the included studies, pain measurement method, main outcomes, quality of evidence and reported limitations.

Each review was classified according to the main procedural block: irrigation, instrumentation, or obturation. If a review addressed more than one potentially relevant factor, classification was based on the primary clinical comparison and the main outcome reported in relation to post-operative pain. Disagreements between the two reviewers were resolved through discussion. If consensus could not be reached, one expert operator was consulted.

A formal kappa statistic was not calculated during the original extraction process; Nevertheless, the use of a pilot calibration phase, duplicate independent extraction and consensus discussion was aimed to reduce misclassification and improve reproducibility.

### 2.7. Quality Assessment

Following a recent umbrella review, the methodological quality of the included systematic reviews was assessed using the AMSTAR-2 (A Measurement Tool to Assess Systematic Reviews 2) checklist [20], a modified version of the original AMSTAR version released in 2007 [23]. AMSTAR 2 was used exclusively to assess the methodological quality and overall confidence of the included systematic reviews, and not used to assess the certainty of the underlying clinical evidence.

Each item was rated as “Yes” if adequately addressed, “Partial Yes” if partially addressed, and “No” if not addressed [24].

The critical domains considered were the following: protocol registration, adequacy of the literature search, justification for excluding individual studies, use of an appropriate method to assess risk of bias in included studies, adequacy of meta-analytical methods, consideration of risk of bias when interpreting results, and assessment of the presence and potential impact of publication bias.

### 2.8. Data Synthesis and Analysis of the Overlap Across Different PIOS

Due to heterogeneity among the included systematic reviews regarding interventions, outcomes, and methodological approaches, a qualitative narrative synthesis of the findings was performed. Where applicable, results from meta-analyses reported in the included systematic reviews were summarized descriptively. To assess the degree of overlap of primary studies across the included systematic reviews, a citation matrix was constructed, listing primary studies in columns and systematic reviews in rows. The corrected covered area (CCA) was then calculated to quantify the extent of overlap as follows.

CCA = (N − R)/[(r × c) − r] × 100

N = is the total number of primary study occurrences across the included systematic reviews;
r = is the number of unique primary studies;
c = is the number of systematic reviews.

Overlap was interpreted as slight (0–5%), moderate (6–10%), high (11–15%), or very high (>15%) according to previous studies [25,26].

The degree of overlap was not used as a formal quantitative weighting factor, as no de novo meta-analysis of primary studies was performed. However, CCA values were used to inform the narrative interpretation of the findings. In domains with moderate overlap, consistency across reviews was interpreted cautiously. In domains with high or very high overlap, concordant findings across reviews were not considered fully independent, as some primary studies may have contributed repeatedly to multiple reviews.

## 3. Results

### 3.1. Study Selection

The search strategy identified 368 records. After removal of 204 duplicates, 164 records were screened by title and abstract. Of these, 113 records were excluded because they were laboratory studies; did not relate to endodontics; did not address the PIOS question; were case series, commentaries, case reports, books, or narrative reviews; were not available through the institution; or did not include a meta-analysis.

The remaining 51 full-text reports were assessed for eligibility. Of these, 26 were excluded because they did not meet the predefined inclusion criteria, mainly because they focused on laser interventions, children, occlusion, single- versus multiple-visit treatment, primary teeth, pharmacological pain management, studies without comparisons, vital pulp therapy, or intracanal medicaments.

A total of 25 systematic reviews and meta-analyses published in English within the selected timeframe met the inclusion criteria and were included in the final analysis [27,28,29,30,31,32,33,34,35,36,37,38,39,40,41,42,43,44,45,46,47,48,49,50,51]. The characteristics of the included systematic reviews are reported in Table 1.

All included articles were systematic reviews with meta-analysis published in peer-reviewed journals. Table 1 shows relevant methodological variability across the included reviews. Protocol registration was reported in most reviews, whereas four reviews did not report registration in PROSPERO or another public database [31,37,39,44]. The number of primary studies included in each review varied substantially, ranging from small meta-analyses based on three or four studies to broader reviews including more than twenty primary studies. Risk-of-bias assessment was performed in all included reviews, but the tools used were not uniform. Most reviews used the Cochrane Risk of Bias tool or RoB 2, whereas one review used the JBI Critical Appraisal Tool for randomized controlled trials. Funding was reported in a minority of reviews, while most reviews declared no funding.

**Figure 1:**
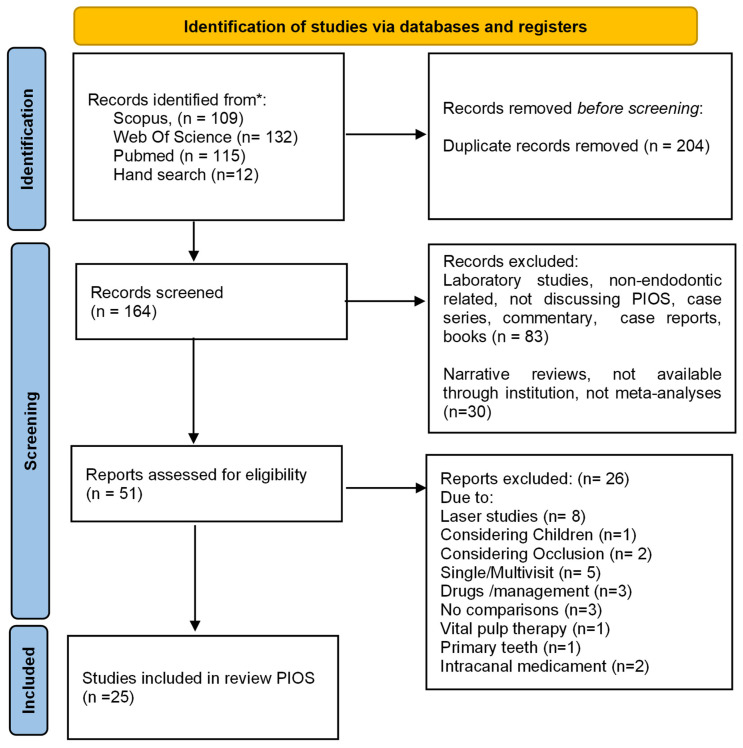
Flowchart of review identification and selection. Twenty six reviews were excluded as they were outside the topic of the PIOS. * Number of records identified from each database or register searched reported (rather than the total number across all databases/registers).

The PRISMA flow diagram (Figure 1) illustrates the selection process, including the reasons of exclusions of assessed reviews.

### 3.2. PIOS 1: POP and Irrigation Protocols

A total of nine systematic reviews investigating the relationship between irrigation protocols and post-operative pain were included. Detailed findings are presented in Table 2. The investigated factors included irrigant concentration, irrigant activation techniques, irrigant type comparison, and intracanal cryotherapy.

Regarding irrigant concentration, two systematic reviews reported a higher prevalence of post-operative pain when higher concentrations of sodium hypochlorite (NaOCl ≥ 5%) were used, compared with lower concentrations (NaOCl 1.0–3.0%).

Concerning irrigation activation techniques, ultrasonic or agitation techniques showed a reduction in post-operative pain at earlier times (6 to 48 h) compared with conventional syringe irrigation. When comparing different irrigants, only one review was published that showed no significant differences in post-operative pain when using NaOCl versus chlorhexidine.

Four systematic reviews evaluated the effect of intracanal cryotherapy, reporting lower pain levels during the early post-operative period (6–24 h) compared with conventional irrigation. One review specifically reported that cryotherapy reduced post-operative pain in teeth with symptomatic apical periodontitis at 24 h. However, these differences tended to disappear at later follow-up times (48–72 h and 7 days).

The data suggests that lower NaOCl concentrations, irrigant activation protocols, and intracanal cryotherapy may be associated with reduced short-term post-operative pain, mainly within the first 24–48 h. However, these findings should be interpreted as recurrent low- to moderate-certainty trends rather than definitive clinical recommendations, because the included reviews differed in pain assessment methods, follow-up periods, and methodological quality.

### 3.3. PIOS 2: POP and Instrumentation Protocols

A total of eight systematic reviews evaluated the influence of instrumentation-related variables on post-operative pain, including apical patency, foraminal enlargement, and different instrumentation systems. Further details are provided in Table 3.

Three systematic reviews investigated apical instrumentation-related factors. Two reviews evaluated apical patency versus non-apical patency, suggesting that maintaining apical patency may reduce or not increase post-operative pain at early time points, particularly at 24 and 48 h, although findings were not consistently statistically significant across studies. One review evaluated foraminal enlargement in necrotic teeth and reported higher post-operative pain during the first post-operative days compared with conventional endodontic treatment.

Five systematic reviews compared different instrumentation systems, including manual, rotary, reciprocating, and mechanical NiTi techniques. Several reviews reported lower incidence or intensity of post-operative pain with rotary systems compared with reciprocating or manual instrumentation. However, the evidence was not fully consistent, as one review reported no significant difference between rotary and reciprocating motions, while another reported lower post-operative pain with reciprocating kinematics.

The data suggest that instrumentation protocols may influence short-term post-operative pain, but the direction of the effect is not uniform across reviews. Rotary systems, particularly multi-file rotary systems, were frequently associated with lower pain outcomes compared with reciprocating or manual techniques, but conflicting findings limit definitive conclusions. Evidence regarding apical patency suggests that maintaining patency does not increase post-operative pain and may reduce early pain. These findings should be interpreted cautiously because of clinical and methodological heterogeneity, including differences in diagnosis, instrumentation protocols, file systems, apical preparation, pain assessment methods, and follow-up periods.

### 3.4. PIOS 3: POP and Obturation Protocols

A total of eight systematic reviews assessed the association between obturation strategies and post-operative pain, including comparisons between different sealer types and obturation materials. Detailed results are summarized in Table 4.

Most systematic reviews focused on comparisons between bioceramic sealers and epoxy resin-based sealers. The majority of the evidence suggested no significant differences in post-operative pain between different sealer types, particularly in the delayed post-operative period (3–7 days).

Some reviews reported slightly lower pain levels with bioceramic sealers during the first 24 h, although these differences were not consistently observed across studies. The most recent review also included bioactive glass-based sealers and reported no significant difference in pain occurrence at 24 h, 48 h, and 7 days compared with epoxy resin-based sealers.

Other comparisons involving zinc oxide–eugenol sealers, calcium silicate-based sealers, and calcium hydroxide-based sealers similarly showed no clinically significant differences in post-operative pain outcomes.

The data does not support a clinically consistent difference in post-operative pain between bioceramic/calcium silicate-based sealers and resin-based sealers, especially at delayed follow-up periods. Some reviews reported lower early pain with bioceramic or calcium silicate-based sealers, but these findings were not uniform. The certainty of evidence ranged from low to moderate, and the findings were frequently limited by risk of bias, small sample sizes, and variability in treatment protocols.

### 3.5. Methodological Quality and Overall Confidence

The methodological quality of the included systematic reviews was assessed using AMSTAR 2, and the results are summarized in Table S2 of the Supplementary Materials.

Overall, the methodological quality was heterogeneous, with several reviews showing methodological limitations. Weaknesses were identified in key AMSTAR 2 domains. Four reviews did not report PROSPERO registration or other public databases. Several reviews only partially fulfilled the AMSTAR 2 criterion regarding predefined methods. Literature search strategies were generally reported but a list of excluded studies with justification was not provided in 11 reviews. Risk-of-bias assessment was performed in all included reviews; however, several reviews only partially satisfied AMSTAR 2 criteria. Risk of bias in the primary studies was not consistently addressed, and publication bias was often not assessed, raising concerns about the overall reliability of the available evidence. Funding sources were inconsistently reported across the reviews.

### 3.6. Overlap Assessment Across PIOS

The degree of overlap varied across the three PIOS domains, and is reported in Table S3 of the Supplementary Material.

For irrigation, nine systematic reviews included 36 unique primary studies and 68 total study occurrences, yielding a CCA of 11.1%, which indicates a high overlap. For instrumentation, eight systematic reviews included 47 unique primary studies and 88 total study occurrences, resulting in a CCA of 12.5%, indicating a high overlap. For obturation, eight systematic reviews included 43 unique primary studies and 96 total study occurrences, with a CCA of 17.6%, indicating a very high overlap. The high and very high overlap suggest that several reviews within these domains relied on substantially shared sets of primary studies. Therefore, findings in these domains should not be interpreted as fully independent confirmation.

## 4. Discussion

Post-operative pain may be influenced by several patient-related and preoperative factors (out of the scope of the present study), including baseline pathological status, systemic health, psychological status, and medication intake.

This umbrella review aimed to consolidate the current evidence on post-operative pain occurrence in relation to the recent advances in endodontics, particularly regarding irrigation, instrumentation and obturation procedures. For these reasons we deliberately restricted the search strategy to the last ten years.

Irrigation strategies were implemented in recent years with new activating devices and techniques, and we tried to reconsider the traditional use and concentration of sodium hypochlorite (NaOCl) and demineralizing agents such as EDTA.

Sodium hypochlorite (NaOCl) is still considered a first choice for root canal treatment thanks to its antimicrobial activity, organic tissue dissolution capacity, high availability and low cost. In the present umbrella review, two included systematic reviews reported a lower prevalence of short-term post-operative pain when lower NaOCl concentrations were used compared with higher concentrations. This finding may be clinically relevant, but it should be interpreted cautiously because the included reviews differed in pain assessment methods, follow-up periods, and reported certainty of evidence.

NaOCl concentration and delivery strategies are particularly relevant to the safety profile of irrigation protocols. Risks of NaOCl extrusions, although rare, are a real emergency in clinical practice, inducing swelling hematoma and necrosis of the surrounding structures [52]. Some micromorphological and vibrational investigations reported collagen degradation and chemical weakening of dentinal structure, potentially leading to unexpected fractures in absence of other factors [53].

New strategies to avoid POP upon irrigant extrusions may include the reduction in concentration of NaOCl (using 1 to 3% concentration) [27] and the implementation of Chlorhexidine (CHX) as a final irrigant in the case of highly infected roots, where the apical morphology is often resorbed or irregular [54]. CHX was proposed as an alternative or as an adjunctive solution to NaOCl primarily for its antimicrobial properties and substantivity, being useful as a final irrigant in endodontic cases with a high bacterial load [55].

However, only one included systematic review compared NaOCl with chlorhexidine in relation to post-operative pain and reported no significant difference between the two irrigants [30]. Therefore, it does not provide sufficient evidence to support CHX as a strategy for reducing post-operative pain.

Cryotherapy was also an interesting aspect less debated in the literature. Four systematic reviews evaluated cryotherapy, generally reporting lower pain levels during the early post-operative period, mainly within 6–24 h, compared with conventional irrigation [32,33,34,47]. These findings contrast with recent literature protocols that suggest the activation of irrigant solutions to improve activity and penetration of the NaOCl in highly infected cases [56], and future studies must investigate the relation between POP and effective cleaning of the root canals using cryotherapy.

Machine-assisted irrigation activation techniques (i.e., sonic irrigation) have been recently standardized in endodontic treatment [16]. Compared to manual techniques such as conventional needle irrigation and manual dynamic activation using files or gutta-percha, these techniques enhance flow and distribution of endodontic irrigants, particularly at the apical third. Therefore, it is important to analyze the relation of machine-assisted irrigation to POP. Interestingly, the studies reported lower pain levels when using machine-assisted irrigation, which may be explained by the higher risk of incorrect irrigation procedures when using manual techniques.

The lower post-operative pain reported with irrigant activation may be related to improved irrigant penetration, enhanced removal of infected debris and better irrigant exchange in the apical third. Controlled activation may also reduce vapor lock effects [57] and may reduce excessive pressure that leads to irrigant extrusion out of the apex [16]. However, these mechanisms were not directly tested in the included reviews and should be interpreted as plausible explanatory hypotheses rather than definitive causal pathways.

Regarding instrumentation techniques, important innovations in endodontics include the use of reciprocating single-file techniques and rotary NiTi systems [18]. These techniques allow for more rapid and efficient instrumentation of the root canal, with lower risks of instrument fracture [18,58]. However, the relation between the reciprocating movements and potential extrusion of apical debris—and consequent POP—has been widely debated, with controversial findings [59].

In the present umbrella review, eight systematic reviews evaluated instrumentation-related variables, including apical patency, foraminal enlargement, and different instrumentation systems. Five reviews compared different instrumentation systems, including manual, rotary, reciprocating, and mechanical NiTi techniques. Overall, several reviews reported lower incidence or intensity of post-operative pain with rotary instrumentation, particularly multi-file rotary systems, compared with reciprocating or manual techniques. However, this trend was not uniform. One review reported no significant difference between rotary and reciprocating motions [48], whereas another reported lower overall post-operative pain with reciprocating kinematics [49].

The association between instrumentation systems and post-operative pain may be related to differences in file kinematics, shaping sequence, number of instruments, taper, cutting efficiency, and apical debris extrusion. However, this interpretation remains indirect because the included reviews did not uniformly isolate the effects of kinematics, taper, apical enlargement, and file design. Apical patency and foraminal enlargement are both related to the management of the apical limit during canal instrumentation and represent distinct clinical approaches.

Maintaining apical patency with a small file may help preserve working length, prevent apical blockage, and facilitate irrigant delivery to the apical portion of the root. However, repeated file passage beyond the apical foramen may cause mechanical irritation and promote extrusion of infected debris or irrigants, potentially increasing periapical inflammation and post-operative pain, particularly in teeth with a higher microbial or inflammatory burden [59]. Foraminal enlargement may amplify these risks because it involves intentional widening of the apical foramen rather than simple maintenance of patency [60]. However, these mechanisms were not directly tested in the included reviews and should be interpreted as plausible explanatory pathways rather than definitive causal mechanisms. Two systematic reviews evaluated apical patency versus non-apical patency, suggesting a possible reduction in post-operative pain at early time points, particularly at 24 and 48 h, when apical patency was maintained [35,36]. In contrast, foraminal enlargement was associated with higher post-operative pain during the first post-operative days compared with conventional endodontic treatment. Preparation protocols using reduced taper may, in theory, influence debris extrusion and post instrumentation flare-up risk [61].

The introduction of hydraulic calcium silicates (HCSC)—and later premixed bioceramics—as root canal sealers led to further innovations in endodontics. HCSC chemicals physical and biological have been widely investigated, supporting their use in close contact with periapical tissues and in complex endodontic situations, as wide apexes, irregular apexes and periapical lesions. Recent clinical studies supported a similar healing and survival outcome compared to conventional epoxy resin-based sealers, but also demonstrated a higher tendency for periapical extrusions with both warm and cold techniques [62,63,64,65].

The relation between POP and hydraulic calcium silicate sealers have been discussed in several systematic reviews. Recently, an umbrella review analyzing all studies in recent years reported that no differences in POP can be expected according to the sealers used, mainly due high heterogeneity of the included reviews and the wide range of materials and time frames [20].

Analogously, our review reported a similar trend even if the time frame was limited to the last 10 years. Taken together, our study also confirms the large heterogeneity of the previous investigations, but shows a slightly lower tendency of POP when hydraulic sealers are used. This claim, however, requires more standardized systematic reviews. It is also important to note that clinical trials on these sealers have only short-term follow-up, due the relatively recent introduction of sealers. Moreover, the relation between POP and cold versus warm techniques cannot be analyzed due to the scarcity of clinical trials associating HCSC or premixed sealers with warm techniques.

Concerning the methodological quality of the included systematic reviews, some variability was observed among studies. Some key methodological domains showed weaknesses, including the absence of protocol registration, incomplete or non-reproducible search strategies, lack of justification for study exclusion, and insufficient consideration of risk of bias when interpreting results. As shown in Table S2, several domains were frequently rated as “No” or “Partial Yes”, indicating methodological limitations that may affect the transparency, reproducibility, and reliability of the available evidence. These issues, together with variability in eligibility criteria, clinical comparisons, outcome assessment, and analytical approaches, contribute to the uncertainty of the findings.

The analysis of the overlap (Table S3) indicates caution in the interpretation of the findings. Irrigation and instrumentation showed a high overlap, with a CCA of 11.1%, whereas obturation showed a very high overlap, with a CCA of 17.6%. This means that several reviews were based on a shared set of primary studies. For this reason, conclusions across all domains were interpreted cautiously and potentially redundant, in the obturation domain.

Some limitations should be considered.

A marked heterogeneity across the included systematic reviews is observed, in variable methodological quality, eligibility criteria, baseline diagnoses, treatment protocols, pain assessment methods, follow-up periods, denominators, and reporting standards.

Most evidence was related to short-term post-operative pain, while long-term data for newer materials and techniques remain limited. The absence of standardized terminology and pain assessment methods across clinical trials could limit the comparability among the studies.

Overlap of primary studies was present and was particularly high in the obturation domain; therefore, agreement across reviews should not necessarily be interpreted as independent confirmation.

Certainty-of-evidence judgments were extracted as reported by the included reviews and were not reassessed de novo using GRADE at the umbrella review level. Therefore, the observed associations should be interpreted as tentative directional patterns from heterogeneous and partially overlapping secondary evidence, rather than as stable comparative clinical conclusions or practice recommendations.

## 5. Conclusions

Within the above-mentioned limits of this umbrella review, lower sodium hypochlorite concentrations, irrigant activation protocols and intracanal cryotherapy were the procedural factors most associated with reduced short-term post-operative pain after non-surgical root canal treatment. Instrumentation protocols may also influence post-operative pain; however, reviews comparing rotary, reciprocating, and manual systems were heterogeneous, and no definitive superiority of a single kinematic approach can be established. No clinically relevant differences between bioceramic/calcium silicate-based sealers and resin-based sealers were observed, but the very high overlap of primary studies in this domain limits interpretation as independent confirmation. These findings should be interpreted as a trend from partially redundant secondary evidence rather than as independent confirmation.

Future high-quality systematic reviews and adequately powered clinical studies, using standardized pain outcomes, clearly reported baseline diagnoses, and consistent follow-up, are needed.

## Figures and Tables

**Table 1:** Characteristics of included systematic reviews.

Study (Author-Year) N	Journal Published	Meta-Analysis	Number of Included Studies	Risk of Bias Tool	Protocol Registered	Source of Funding
Prasad et al. (2024) [27]	*Journal of Endodontics*	Yes	5	Cochrane’s RoB 2	PROSPERO	NO
Sabino-Silva et al. (2023) [28]	*Clinical Oral Investigations*	Yes	10 for SR 8 for MA	Cochrane’s RoB 2	PROSPERO	NO
Chalub et al. (2022) [29]	*Clinical Oral Investigations*	Yes	8 for SR 4 for MA	Cochrane’s RoB	PROSPERO	YES
Martins et al. (2021) [30]	*Indian Journal of Dental Research*	Yes	3	Cochrane’s RoB	PROSPERO	NO
Decurcio et al. (2019) [31]	*Journal of Endodontics*	Yes	6 for RA 3 for MA	Cochrane’s RoB 2	None	NO
Monteiro et al. (2020) [32]	*Clinical Oral Investigations*	Yes	8 for SA 6 for MA	Cochrane’s RoB	PROSPERO	NO
Almohaimede et al. (2021) [33]	*Int. J. Environ. Res. Public Health*	Yes	16 for RA 9 for MA	Cochrane’s RoB 2	PROSPERO	NO
Sadaf et al. (2020) [34]	*Journal of Endodontics*	Yes	8 for SR 6 for MA	Cochrane’s RoB	PROSPERO	NO
Liu et al. (2024) [35]	*European Journal of Oral Science*	Yes	12	Cochrane’s RoB 2	PROSPERO	NO
Abdulrab et al. (2018) [36]	*Journal of Endodontics*	Yes	5 for SR 4 for MA	Cochrane’s Rob	PROSPERO	NO
Sun et al. (2018) [37]	*Oral Diseases*	Yes	17 for SR 10 for MA	Cochrane’s RoB	None	YES
Silveira et al. (2021) [38]	*Iranian Endodontic Journal*	Yes	21 for SR 17 for MA	Cochrane’s RoB	PROSPERO	NO
Hou et al. (2017) [39]	*BMC Oral Health*	Yes	3	Cochrane’s RoB	None	YES
Zamparini et al. (2024) [40]	*International Endodontic Journal*	Yes	15	Cochrane’s RoB 2	PROSPERO	NO
Seron et al. (2023) [41]	*Odontology*	Yes	18 for SR 17 MA	Cochrane’s RoB 2	PROSPERO	YES
Monteiro et al. (2022) [42]	*Restorative Dentistry & Endodontics*	Yes	17 for SR 15 MA	Cochrane’s RoB 2	PROSPERO	NO
Sponchiado Junior et al. (2021) [43]	*European Journal of Dentistry*	Yes	5 for RA 3 for MA	JBI Critical Appraisal Tool for RCTs	PROSPERO	YES
Jamali et al. (2021) [44]	*Pesqui. Bras. Odontopediatria Clín. Integr.*	Yes	4	Cochrane’s RoB	None	NO
Chopra et al. (2022) [45]	*Journal of Functional Biomaterials*	Yes	12 for SR 8 for MA	Cochrane’s RoB 2	PROSPERO	NO
Mekhdieva et al. (2021) [46]	*Journal of Clinical Medicine*	Yes	9	Cochrane’s RoB 2	PROSPERO	NO
Hespanhol et al. (2022) [47]	*Restorative Dentistry & Endodontics*	Yes	8 for SR 4 for MA	Cochrane’s RoB	PROSPERO	YES
Rabani Nobar et al. (2021) [48]	*European Endodontic Journal*	Yes	19 for SR 15 for MA	Cochrane’s RoB	PROSPERO	NO
Martins et al. (2019) [49]	*Journal of Conservative Dentistry*	Yes	10 for SR 5 for MA	Cochrane’s RoB	PROSPERO	NO
Borges Silva et al. (2017) [50]	*Journal of Endodontics*	Yes	5 for SR 3 for MA	Cochrane’s RoB	PROSPERO	NO
Hegde et al. (2025) [51]	*Journal of Conservative Dentistry and Endodontics*	Yes	23 for SR 11 for MA	Cochrane’s RoB 2	PROSPERO	NO

**Table 2:** POP and irrigation.

Study (Author-Year) N	Groups	Sample Size/Unit Reported	Search Period	Pain Assessment Methods	Results	Reported Certainty of Evidence	Preoperative Diagnosis	Comments/ Limitations
**Irrigant concentration**								
Prasad et al. (2024) [27]	NaOCl (≤3%) vs. (≥5%)	674 patients	2018–2022	Binary classification (Y/N) VAS NRS	POP was twice as likely to occur with high concentrations of NaOCl than with low concentrations.	LOW	Pulpitis Necrosis with AP Chronic AP	Clinical and methodological heterogeneity among the include studies.
Sabino-Silva et al. (2023) [28]	NaOCl LC (0.5–3%) vs. HC (≥5%)	1021 teeth	2010–2021	VAS NRS Unspecific questionnaire	Lower prevalence of pain in LC versus HC. No differences at 7 days post-treatment.	VERY LOW	Necrosis with AP NR	Reduced number of studies that assessed the outcome and difficulties in to the assessment of post-operative pain.
**Irrigant activation**								
Chalub et al. (2022) [29]	UI vs. CI	554 patients	2015–2020	VAS Incidence of pain	UI had lower POP (6, 24, 48 h) when compared to the CI.	MODERATE (24–48 h–7 days) LOW (6–72 h)	Pulpitis Pulp necrosis Chronic AP	Low number of studies and heterogeneity.
Martins et al. (2021) [30]	NaOCl vs. CHX	254 patients	2010–2015	VAS 4 point scale	No differences in POP using NaOCl and CHX.	LOW	Pulpitis Necrosis with AP Chronic AP	Small number of studies.
**Irrigant comparison**								
Decurcio et al. (2019) [31]	Agitation vs. syringe irrigation	758 patients	2010–2018	VAS	Machine-assisted agitation resulted in less PP compared with syringe irrigation with needle at 24 and 48 h.	MODERATE	Pulpitis Vital pulp Chronic AP Necrosis with AP	Heterogeneity among the included studies.
**Cryotherapy**								
Monteiro et al. (2020) [32]	Cryotherapy vs. conventional irrigation	158 patients	2016–2019	VAS	Lower pain levels at 6 and 24 h for cryotherapy. No differences at 48 and 72 h.	VERY LOW	Pulpitis Necrosis with AP	Variability among the studies (methodology), small number of studies.
Almohaimede et al. (2021) [33]	Cryotherapy vs. conventional irrigation	1032 patients	2016–2021	VAS HEFT-PARKER SCALE	Reduction in PP at 6, 24, 48 and 72 h after treatment. No differences at 7 days.	LOW	Vital pulp Pulpitis Necrosis with AP Chronic AP	Serious concerns about the risk of bias and inconsistency; study heterogeneity.
Sadaf et al. (2020) [34]	Cryotherapy vs. conventional irrigation	810 patients	2016–2019	VAS HP VAS	Reduction in POP at 6 and 24 h. No differences at 48, 72 h and 7 days.	MODERATE	Vital pulp Pulpitis Necrosis with AP	The effect of intracanal cold irrigation might be more pronounced in patients with apical periodontitis and in patients with severe preoperative pain.
Hespanhol et al. (2022) [47]	Intracanal cryotherapy vs. conventional irrigation	980 patients	2016–2019	VAS	Cryotherapy reduced POP in teeth with SAP at 24 h; no association in teeth with normal periapical tissue at 24/48 h.	VERY LOW	Vital pulp Pulpitis Necrosis with AP Symptomatic AP	Risk of bias, heterogeneity and small samples; variability in temperature, volume and application time.

**Table 3:** POP and instrumentation.

Study (Author-Year) N	Groups	Sample Size/Unit Reported	Search Period	Pain Assessment Method	Results	Reported Certainty of Evidence	Preoperative Diagnosis	Comments/ Limitations
Apical patency/Foraminal enlargement								
Liu et al. (2024) [35]	Apical patency vs. Non-apical patency	1737 patients	2009–2023	VAS NRS PAIN SCORES Not mentioned	AP significantly reduced post-operative pain at 24 and 48 h after treatment, but no differences were observed at 72 h and 7 days.	LOW TO MODERATE	Pulpitis Necrosis with AP Chronic AP	High protocol heterogeneity among the studies.
Abdulrab et al. (2018) [36]	Apical patency vs. Non-apical patency	721 patients	2009–2018	MODIFIED VAS ORDINAL SCALE PAIN SCALE	AP resulted in less post-operative pain compared with NAP, but the difference was not statistically significant.	MODERATE	Vital pulp Necrotic pulp (with/without AP) NR	Heterogeneity, statistics based on small number of studies.
Borges Silva et al. (2017) [50]	Foraminal enlargement vs. conventional endodontic treatment	331 teeth	1987–2017	VAS Simple verbal categorization	FE was associated with higher POP in the first days; no significant difference in analgesic use, flare-up or swelling.	LOW	Necrosis with AP	Heterogeneous protocols; unclear/high RoB in some studies; subgroup/sensitivity analyses not possible.
Instrumentation systems								
Sun et al. (2018) [37]	Rotary, manual and reciprocating instrumentation systems	1415 teeth	2002–2017	VAS VRS FUNCTIONAL PAIN SCALE	Multi step rotary instruments had lower incidence and intensity of POP compared to hand files and reciprocating systems.	LOW	NR	Presence of non-randomized trials. Blinding not feasible. It was not possible to assess publication bias.
Silveira et al. (2021) [38]	Reciprocating vs. rotary instrumentations systems	2816 patients	2012–2020	VAS VRS PAIN INTENSITY NRS FUNCTIONAL PAIN SCALE	Rotary systems showed lower incidence of POP	LOW	Pulpitis Necrosis with AP Chronic AP	Clinical and methodological variations among the included studies.
Hou et al. (2017) [39]	Rotary vs. reciprocating instrumentation systems	1317 patients	2013–2015	VAS	Rotary instrument is associated with a lower incidence of POP than reciprocating instruments	LOW	Not specified	Heterogeneity, Inconsistent instrumentation protocol.
Rabani Nobar et al. (2021) [48]	Rotary vs. reciprocating instrumentation systems	2767 teeth (2394 in MA)	2015–2019	VAS NRS/VRS Pain scales	No difference in POP at 12, 24 or 48 h; no difference in analgesic intake	LOW	Pulpitis Necrosis with/without AP Retreatment	Clinical/methodological heterogeneity; some high/unclear RoB.
Martins et al. (2019) [49]	Reciprocating vs. rotary instrumentation systems	1442 patients 2047 teeth	2015–2017	VAS Pain scales	Reciprocating systems showed lower overall POP; less severe pain at 48 h than rotary	LOW	Pulpitis Necrosis AP	Heterogeneity in protocols.

**Table 4:** POP and obturation.

Study (Author-Year) N	Groups	Sample Size/Unit Reported	Search Period	Pain Assessment Method	Results	Reported Certainty of Evidence	Preoperative Diagnosis	Comments/ Limitations
Sealer typologies								
Zamparini et al. (2024) [40]	BCS vs. ERBS	1751 patients	2018–2023	VAS NRS LIKERT SCALE	No significant differences in POP for immediate (24 h) and delayed period (3 to 7 days).	MODERATE-HIGH	Pulpitis Necrosis with AP Chronic AP	Limitations in reporting short-term duration
Seron et al. (2023) [41]	BCS vs. ERBS	1294 patients	2018–2023	VAS NRS VDS LIKERT SCALE	BCS reduced POP after 24 h and showed less apical extrusion compared to the ERBS.	MODERATE	Pulpitis Pulp necrosis (with or without AP)	Methodological variability
Monteiro et al. (2022) [42]	BCS vs. ERBS vs. ZOE vs. CaOH	1319	1985–2020	VAS NRS	No difference in POP between sealers.	LOW TO MODERATE	Vital pulp Non-vital pulp	High risk of bias and imprecision
Sponchiado Junior et al. (2021) [43]	CSBS vs. ERBS	421 patients	2018–2020	VAS LIKERT SCALE	No significant difference at 24 h or 48 h. CSBS had lower pain intensity only from 48 h.	MODERATE	Pulpitis Necrosis with AP Chronic AP	Variability among studies about protocols adopted
Jamali et al. (2021) [44]	RBS vs. BCS	121 patients	2017–2020	VAS	No statistically significant differences between groups.	HIGH	Not reported	A larger sample size is required
Chopra et al. (2022) [45]	RBS vs. CSBS	833 teeth	2013–2021	VAS MODIFIED	No clinical differences.	LOW TO MODERATE	Pulpitis Necrosis with AP Chronic AP Root resorption	Heterogeneity about protocols
Mekhdieva et al. (2021) [46]	BCS vs. RBS	426 teeth	2018–2020	VAS VRS HEFT-PARKER PAIN RATING SCALE	No differences in POP.	LOW	Pulp/periapical pathologies Symptomatic/non	Inter-study variability and inconsistency, lack of clinically relevant outcomes
Hegde et al. (2025) [51]	CSBS/BGS vs. ERBS	2581 patients	2018–2024	VAS NRS/Numerical scales VDS PAI/radiographs for healing	No significant difference in pain occurrence at 24 h, 48 h and 7 days; CSBS comparable to ERBS, may improve healing.	NR	Pulpitis Necrosis with AP Symptomatic/asymptomatic cases Retreatment	Methodological differences and heterogeneity; inconsistent study findings; need uniform confirmatory clinical trials

BCS = bioceramic sealer; ERBS = epoxy resin-based sealer; ZOE = Zinc Oxide Eugenol sealer; CSBS = calcium silicate-based sealers; CaOH = calcium hydroxide-based sealers; BGS = Bioglass containing sealer.

## Data Availability

The study protocol is available in PROSPERO.

## Supplementary Materials

The following supporting information can be downloaded at https://www.mdpi.com/article/10.3390/jcm15124775/s1, File S1: PRISMA_2020_abstract_checklist and PRISMA_2020_checklist; Table S1: Complete database-specific search strategy used to identify systematic reviews and meta-analyses; Table S2: AMSTAR 2 tool; Table S3: CCA among the 3 different PIOS; Table S4: Raw database used to calculate CCA.
