# Supplementary material for: Post-Operative Pain After Endodontic Instrumentation, Irrigation and Obturation: An Umbrella Review of Systematic Reviews Published from 2016 to 2025
Source: J Clin Med. 2026 Jun 19;15(12):4775. doi: 10.3390/jcm15124775 (PMC13301676; doi:10.3390/jcm15124775)
Supplement: Supplementary file 1 [file jcm-15-04775-s001.zip › jcm-4267477-Tables S1-S3.pdf]

SUPPLEMENTARY MATERIALS

Post-Operative Pain After Endodontic Instrumentation, Irrigation and Obturation: An Umbrella Review of Systematic Reviews Published from 2016 to 2025

Table S1	Complete database-specific search strategy used to identify systematic reviews and meta-analyses.
**Database**	**Search strategy, limits, and records retrieved**
**PubMed**	("Pain"[Mesh] OR "Pain, Postoperative"[Mesh] OR pain[Title/Abstract] OR "postoperative pain"[Title/Abstract] OR "post-operative pain"[Title/Abstract] OR "post operative pain"[Title/Abstract] OR "postendodontic pain"[Title/Abstract] OR "post-endodontic pain"[Title/Abstract] OR "post endodontic pain"[Title/Abstract] OR "postobturation pain"[Title/Abstract] OR "post-obturation pain"[Title/Abstract] OR "post obturation pain"[Title/Abstract] OR "interappointment pain"[Title/Abstract] OR "inter-appointment pain"[Title/Abstract] OR "flare-up"[Title/Abstract] OR "flare up"[Title/Abstract] OR flareup[Title/Abstract] OR "analgesic consumption"[Title/Abstract] OR "analgesic intake"[Title/Abstract] OR "symptomatic apical periodontitis"[Title/Abstract])<br>AND<br>("Endodontics"[Mesh] OR "Root Canal Therapy"[Mesh] OR "Dental Pulp Diseases"[Mesh] OR "Periapical Diseases"[Mesh] OR endodontic*[Title/Abstract] OR endodont*[Title/Abstract] OR "root canal"[Title/Abstract] OR "root canal treatment"[Title/Abstract] OR "root canal therapy"[Title/Abstract] OR "root canal preparation"[Title/Abstract] OR "non-surgical endodontic"[Title/Abstract] OR "nonsurgical endodontic"[Title/Abstract])<br>AND<br>("Root Canal Irrigants"[Mesh] OR "Sodium Hypochlorite"[Mesh] OR "Chlorhexidine"[Mesh] OR "Root Canal Filling Materials"[Mesh] OR "Dental Instruments"[Mesh] OR irrigat*[Title/Abstract] OR irrigant*[Title/Abstract] OR "sodium hypochlorite"[Title/Abstract] OR NaOCl[Title/Abstract] OR chlorhexidine[Title/Abstract] OR CHX[Title/Abstract] OR EDTA[Title/Abstract] OR "irrigant activation"[Title/Abstract] OR activation[Title/Abstract] OR activated[Title/Abstract] OR ultrasonic[Title/Abstract] OR sonic[Title/Abstract] OR agitation[Title/Abstract] OR cryotherapy[Title/Abstract] OR "cold irrigation"[Title/Abstract] OR instrument*[Title/Abstract] OR instrumentation[Title/Abstract] OR "canal shaping"[Title/Abstract] OR shaping[Title/Abstract] OR rotary[Title/Abstract] OR reciprocating[Title/Abstract] OR manual[Title/Abstract] OR "hand file"[Title/Abstract] OR "hand files"[Title/Abstract] OR "apical patency"[Title/Abstract] OR "apical enlargement"[Title/Abstract] OR "apical preparation"[Title/Abstract] OR taper[Title/Abstract] OR obturat*[Title/Abstract] OR obturation[Title/Abstract] OR "root canal filling"[Title/Abstract] OR sealer*[Title/Abstract] OR "endodontic sealer"[Title/Abstract] OR "endodontic sealers"[Title/Abstract] OR bioceramic*[Title/Abstract] OR "calcium silicate"[Title/Abstract] OR "calcium silicate-based"[Title/Abstract] OR "epoxy resin"[Title/Abstract] OR "resin-based"[Title/Abstract] OR "gutta-percha"[Title/Abstract] OR "intracanal medicament"[Title/Abstract] OR "intracanal medicaments"[Title/Abstract]) AND<br>("Systematic Review"[Publication Type] OR "Meta-Analysis"[Publication Type] OR "systematic review"[Title/Abstract] OR "systematic reviews"[Title/Abstract] OR "meta-analysis"[Title/Abstract] OR "meta analysis"[Title/Abstract] OR metaanalysis[Title/Abstract] OR "network meta-analysis"[Title/Abstract] OR "network meta analysis"[Title/Abstract] OR "umbrella review"[Title/Abstract])<br><br>Limits applied: publication date 2016–2025; English language; article type/systematic review or meta-analysis where applicable. Search date: 11 May 2026. Records retrieved before deduplication: n = 115.
**Scopus**	TITLE-ABS-KEY<br>((pain OR "postoperative pain" OR "post-operative pain" OR "post operative pain" OR "postendodontic pain" OR "post-endodontic pain" OR "post endodontic pain" OR "postobturation pain" OR "post-obturation pain" OR "post obturation pain" OR "interappointment pain" OR "inter-appointment pain" OR "flare-up" OR "flare up" OR flareup OR "analgesic consumption" OR "analgesic intake" OR "symptomatic apical periodontitis") AND (endodontic* OR endodont* OR "root canal" OR "root canal treatment" OR "root canal therapy" OR "root canal preparation" OR "non-surgical endodontic" OR "nonsurgical endodontic") AND (irrigat* OR irrigant* OR "sodium hypochlorite" OR NaOCl OR chlorhexidine OR CHX OR EDTA OR "irrigant activation" OR activation OR activated OR ultrasonic OR sonic OR agitation OR cryotherapy OR "cold irrigation" OR instrument* OR instrumentation OR "canal shaping" OR shaping OR rotary OR reciprocating OR manual OR "hand file" OR "hand files" OR "apical patency" OR "apical enlargement" OR "apical preparation" OR taper OR obturat* OR obturation OR "root canal filling" OR sealer* OR "endodontic sealer" OR "endodontic sealers" OR bioceramic* OR "calcium silicate" OR "calcium silicate-based" OR "epoxy resin" OR "resin-based" OR "gutta-percha" OR "intracanal medicament" OR "intracanal medicaments") AND ("systematic review" OR "systematic reviews" OR "meta-analysis" OR "meta analysis" OR metaanalysis OR "network meta-analysis" OR "network meta analysis" OR "umbrella review"))<br><br>Records retrieved: n = 109; date of search: 11/05/2026.
**Web of Science**	TS =((pain OR "postoperative pain" OR "post-operative pain" OR "post operative pain" OR "postendodontic pain" OR "post-endodontic pain" OR "post endodontic pain" OR "postobturation pain" OR "post-obturation pain" OR "post obturation pain" OR "interappointment pain" OR "inter-appointment pain" OR "flare-up" OR "flare up" OR flareup OR "analgesic consumption" OR "analgesic intake" OR "symptomatic apical periodontitis") AND (endodontic* OR endodont* OR "root canal" OR "root canal treatment" OR "root canal therapy" OR "root canal preparation" OR "non-surgical endodontic" OR "nonsurgical endodontic") AND (irrigat* OR irrigant* OR "sodium hypochlorite" OR NaOCl OR chlorhexidine OR CHX OR EDTA OR "irrigant activation" OR activation OR activated OR ultrasonic OR sonic OR agitation OR cryotherapy OR "cold irrigation" OR instrument* OR instrumentation OR "canal shaping" OR shaping OR rotary OR reciprocating OR manual OR "hand file" OR "hand files" OR "apical patency" OR "apical enlargement" OR "apical preparation" OR taper OR obturat* OR obturation OR "root canal filling" OR sealer* OR "endodontic sealer" OR "endodontic sealers" OR bioceramic* OR "calcium silicate" OR "calcium silicate-based" OR "epoxy resin" OR "resin-based" OR "gutta-percha" OR "intracanal medicament" OR "intracanal medicaments") AND ("systematic review" OR "systematic reviews" OR "meta-analysis" OR "meta analysis" OR metaanalysis OR "network meta-analysis" OR "network meta analysis" OR "umbrella review"))<br><br>Records retrieved: n = 132; date of search: 11/05/2026.

Table S2	AMSTAR 2 tool															
	1	2	3	4	5	6	7	8	9	10	11	12	13	14	15	16
[27] Prasad N. et al. (2024)	Y	Y	Y	PY	Y	Y	Y	Y	Y	N	Y	Y	Y	Y	N	Y
[28] Sabino-Silva R. et al. (2023)	Y	Y	Y	Y	Y	Y	PY	Y	Y	N	Y	Y	Y	Y	N	Y
[29] Chalub LO et al. (2022)	Y	Y	Y	Y	Y	Y	N	Y	PY	Y	Y	Y	Y	Y	Y	Y
[30] Martins CM et al. (2021)	Y	Y	Y	PY	Y	Y	Y	Y	PY	N	Y	Y	Y	Y	N	Y
[31] Decurcio DA et al. (2019)	Y	PY	Y	PY	Y	Y	Y	Y	PY	N	Y	Y	Y	Y	N	Y
[32] Monteiro LPB et al. (2020)	Y	Y	Y	PY	Y	Y	Y	Y	PY	N	Y	Y	Y	Y	N	Y
[33] Almohaimede A et al. (2021)	Y	Y	Y	PY	Y	Y	Y	Y	PY	N	Y	Y	Y	Y	Y	Y
[34] Sadaf D et al. (2020)	Y	Y	Y	Y	Y	Y	Y	Y	PY	N	Y	Y	Y	Y	Y	Y
[35] Xiquian L et al. (2024)	Y	Y	Y	Y	Y	Y	N	Y	PY	N	Y	Y	Y	Y	N	Y
[36] Abdulrab S et al. (2018)	Y	PY	Y	PY	Y	Y	N	Y	PY	N	Y	Y	Y	Y	N	Y
[37] Sun C. et al. (2018)	Y	PY	Y	PY	Y	Y	N	Y	PY	N	Y	Y	Y	Y	N	Y
[38] da Silveira MT et al. (2021)	Y	Y	Y	PY	Y	Y	N	Y	PY	N	Y	Y	Y	Y	N	Y
[39] Hou XM et al. (2017)	Y	PY	Y	PY	Y	N	Y	Y	PY	Y	Y	Y	Y	Y	Y	Y
[40] Zamparini F et al. (2024)	Y	Y	Y	Y	Y	Y	Y	Y	PY	N	Y	Y	Y	Y	Y	Y
[41] Seron MA et al. (2023)	Y	Y	Y	Y	Y	Y	N	Y	PY	Y	Y	Y	Y	Y	Y	Y
[42] Monteiro CMC et al. (2022)	Y	Y	Y	Y	Y	Y	N	Y	PY	N	Y	Y	Y	Y	Y	Y
[43] Sponchiado Junior EC et al. (2021)	Y	Y	Y	PY	Y	Y	Y	Y	PY	Y	Y	Y	Y	Y	Y	Y
[44] Jamali S et al. (2021)	Y	PY	Y	PY	Y	Y	N	Y	PY	N	Y	Y	N	Y	N	Y
[45] Chopra et al. (2022)	Y	Y	PY	PY	Y	Y	N	Y	PY	N	Y	Y	Y	Y	Y	Y

[46] Mekhdieva E. et al. (2021)	Y	Y	Y	PY	Y	Y	N	PY	PY	N	Y	Y	Y	Y	Y	Y
[47] Hespanhol et al. (2022)	Y	Y	N	Y	Y	Y	PY	Y	Y	N	Y	N	Y	Y	PY	Y
[48] Nobar et al. (2021)	Y	Y	N	PY	Y	Y	Y	Y	Y	N	Y	Y	PY	Y	N	Y
[49] Martins et al. (2019)	Y	Y	N	PY	Y	N	PY	Y	Y	N	Y	N	N	PY	PY	Y
[50] Borges Silva et al. (2017)	Y	Y	N	Y	Y	PY	N	Y	Y	N	Y	Y	Y	Y	N	PY
[51] Hegde et al. (2025)	Y	Y	N	PY	Y	Y	PY	Y	Y	N	Y	N	N	PY	N	Y

Y= Yes PY= Partial Yes N= No

Note: 1. Did the research questions and inclusion criteria for the review include the components of PICO?; 2. Did the report of the review contain an explicit statement that the review methods were established prior to the conduct of the review and did the report justify any significant deviations from the protocol?; 3. Did the review authors explain their selection of the study designs for inclusion in the review?; 4. Did the review authors use a comprehensive literature search strategy?; 5. Did the review authors perform study selection in duplicate?;6. Did the review authors perform data extraction in duplicate?; 7. Did the review authors provide a list of excluded studies and justify the exclusions?
8. Did the review authors describe the included studies in adequate detail?; 9. Did the review authors use a satisfactory technique for assessing the risk of bias (RoB) in individual studies that were included in the review?;10. Did the review authors report on the sources of funding for the studies included in the review?; 11. If meta-analysis was performed, did the review authors use appropriate methods for statistical combination of results?; 12. If meta-analysis was performed, did the review authors assess the potential impact of RoB in individual studies on the results of the meta-analysis or other evidence synthesis?; 13. Did the review authors account for RoB in primary studies when interpreting/discussing the results of the review?;14. Did the review authors provide a satisfactory explanation for, and discussion of, any heterogeneity observed in the results of the review?; 15. If they performed quantitative synthesis did the review authors carry out an adequate investigation of publication bias (small study bias) and discuss its likely impact on the results of the review?;16. Did the review authors report any potential sources of conflict of interest, including any funding they received for conducting the review?

**Table S3**	**CCA among the 3 different PIOS**				
Domains	Reviews	Unique studies	Total study occurrences	CCA (%)	Overlap
PIOS 1 Irrigation	9	36	68	11.1	High
PIOS 2 Instrumentation	8	47	88	12.5	High
PIOS 3 Obturation	8	43	96	17.6	Very high
